# Sharing Annotated Audio Recordings of Clinic Visits With Patients—Development of the Open Recording Automated Logging System (ORALS): Study Protocol

**DOI:** 10.2196/resprot.7735

**Published:** 2017-07-06

**Authors:** Paul J Barr, Michelle D Dannenberg, Craig H Ganoe, William Haslett, Rebecca Faill, Saeed Hassanpour, Amar Das, Roger Arend, Meredith C Masel, Sheryl Piper, Haley Reicher, James Ryan, Glyn Elwyn

**Affiliations:** ^1^ The Dartmouth Institute for Health Policy and Clinical Practice Geisel School of Medicine Dartmouth College Lebanon, NH United States; ^2^ Department of Biomedical Data Science Geisel School of Medicine Dartmouth College Lebanon, NH United States; ^3^ Department of Computer Science Dartmouth College Hanover, NH United States; ^4^ Department of Epidemiology Geisel School of Medicine Dartmouth College Lebanon, NH United States; ^5^ Patient Partner Hanover, NH United States; ^6^ Oliver Center for Patient Safety and Quality Healthcare University of Texas Medical Branch Galveston, TX United States; ^7^ Patient Partner Ludington, MI United States; ^8^ Dartmouth College Hanover, NH United States; ^9^ Ryan Family Practice Ludington, MI United States

**Keywords:** audiovisual aids, patient engagement, machine learning, disease management, caregivers, patients

## Abstract

**Background:**

Providing patients with recordings of their clinic visits enhances patient and family engagement, yet few organizations routinely offer recordings. Challenges exist for organizations and patients, including data safety and navigating lengthy recordings. A secure system that allows patients to easily navigate recordings may be a solution.

**Objective:**

The aim of this project is to develop and test an interoperable system to facilitate routine recording, the Open Recording Automated Logging System (ORALS), with the aim of increasing patient and family engagement. ORALS will consist of (1) technically proficient software using automated machine learning technology to enable accurate and automatic tagging of in-clinic audio recordings (tagging involves identifying elements of the clinic visit most important to patients [eg, treatment plan] on the recording) and (2) a secure, easy-to-use Web interface enabling the upload and accurate linkage of recordings to patients, which can be accessed at home.

**Methods:**

We will use a mixed methods approach to develop and formatively test ORALS in 4 iterative stages: case study of pioneer clinics where recordings are currently offered to patients, ORALS design and user experience testing, ORALS software and user interface development, and rapid cycle testing of ORALS in a primary care clinic, assessing impact on patient and family engagement. Dartmouth’s Informatics Collaboratory for Design, Development and Dissemination team, patients, patient partners, caregivers, and clinicians will assist in developing ORALS.

**Results:**

We will implement a publication plan that includes a final project report and articles for peer-reviewed journals. In addition to this work, we will regularly report on our progress using popular relevant Tweet chats and online using our website, www.openrecordings.org. We will disseminate our work at relevant conferences (eg, Academy Health, Health Datapalooza, and the Institute for Healthcare Improvement Quality Forums). Finally, Iora Health, a US-wide network of primary care practices (www.iorahealth.com), has indicated a willingness to implement ORALS on a larger scale upon completion of this development project.

**Conclusions:**

Upon the completion of this project we will have developed a novel recording system that will be ready for large-scale testing. Our long-term goal is for ORALS to seamlessly fit into a clinic’s and patient’s daily routine, increasing levels of patient engagement and transparency of care.

## Introduction

### Background

Higher recall of medical information is associated with improved disease management, treatment adherence, and higher patient satisfaction; however, recall of medical information is often low, with 40% to 80% of medical information from a clinical visit forgotten immediately by patients [1-5]. Poor knowledge of medical conditions has been identified as a significant barrier to self-management of health conditions associated with lower health status [6]. Difficulty recalling health information is amplified when patients are emotionally charged [7-10]. Lack of health literacy—the ability to perform basic reading and numerical tasks required to function in a health care environment [10]—exacerbates the challenge of patient recall and understanding of health information and affects 59% of adults over 65 years in the United States [11]. Low health literacy is associated with a reduction in the ability of patients to self-manage and interpret health messages and medication labels [12-16]. When patients find it difficult to comprehend health care information during the visit, they are in turn less able to recall information following the visit [17-19].

There have been several advances toward addressing this information recall problem [20]. Providing patients with an after visit summary (AVS) within 3 days of the clinic visit is a key requirement of Meaningful Use of an electronic health record (EHR), stage 1 [21]. Allowing patients to have access to clinician notes through OpenNotes after the visit makes patients feel more in control of their care and improves information recall and medication adherence [22]. Patients also share these notes with family members, which can enhance decision-making skills and care because families can better support a patient through difficult decisions and treatments when they have all the necessary medical information [23]. Note-taking during the visit or specific written instructions postvisit are other methods proposed to improve recall and improve treatment engagement [24-26]. However, strategies dependent on written material are much less effective when the patient has basic health literacy [17,27-29], and the act of note-taking can be distracting to both the patient and the physician.

An alternative approach, based on 40 years of research, is to share audio or video recordings of clinic visits. Patients value listening to and sharing recordings of clinic visits with their families; access to recordings leads to increased patient and family engagement, understanding, recall of health care information, and treatment adherence and reduced decisional regret [8,30-45]. This is also a benefit for caregivers, who are better prepared to provide care, which could in turn reduce caregiver morbidity associated with a perceived lack of self-efficacy related to the provision of care [46,47].

Despite evidence of benefit, patients are rarely offered recordings, yet demand is high as evidenced by the rise of patients secretly recording clinic visits [32,48]. In a recent study of 130 respondents from the general public in the United Kingdom, 15% reported covertly recording a clinic visit, and 77% would like their clinic to offer recordings [49]. The main motivation for recording was to improve understanding and involve family members in care, and the patients who did record reported greater engagement and empowerment. However, patients felt that the absence of a safe, secure, and efficient recording system was a significant barrier. Navigating lengthy recordings was also considered a problem because getting the benefit “depends on picking out...the crucial points...” [32]. A secure system that allows patients to easily navigate recordings by tagging elements of the clinic visit that are most important to them (eg, diagnosis, treatment plan) may be a solution.

### Aims

The purpose of this project is to develop and test an interoperable system to facilitate routine tagged recordings—Open Recording Automated Logging System (ORALS)—with the aim of increasing patient and family engagement in care. ORALS will consist of 2 key elements: (1) technically proficient software using automated machine learning technology to enable accurate and automatic tagging of in-clinic audio recordings and (2) a secure, easy-to-use Web interface enabling the upload and accurate linkage of recordings to patients that can be accessed at home. Our team consists of a range of stakeholders including the Informatics Collaboratory for Design, Development, and Dissemination (ic3d) at Dartmouth; Geisel School of Medicine at Dartmouth; patients; caregivers; and clinicians.

The expected outcome of this project is the development of a widely accepted and scalable recording system, ORALS, that can be used by patients and their families, leading to higher patient and family engagement.

**Figure 1:**
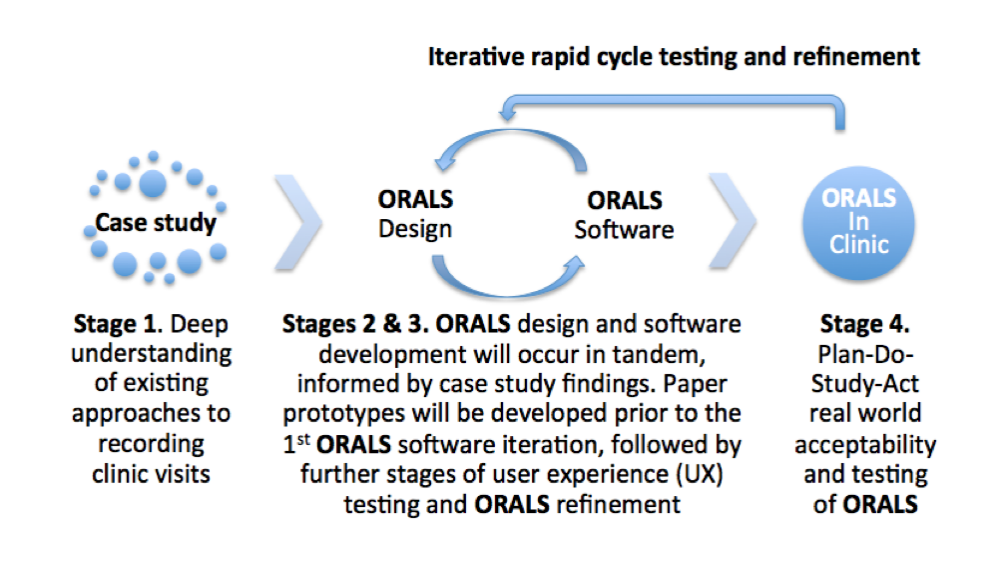
Stages of Open Recording Automated Logging System development.

We will use a mixed methods and agile software development approach, which involves the early and frequent engagement of end-users, to develop and conduct formative testing of ORALS in 4 iterative stages: a case study of pioneer clinics where recordings are currently offered to patients, ORALS design and user experience testing, the development of ORALS software and user interface, and rapid cycle testing of ORALS in a primary care clinic (Figure 1).

## Methods

### Stage 1: Case Study

#### Overview

To gain a deeper understanding of phenomena in their context of use, a case study methodology is recommended [50]. The case study will be guided by Yin’s [51,52] approach, using 5 components: research questions, propositions or purpose, units of analysis, determination of how the data are linked to the propositions, and criteria to interpret the findings. The first step to developing ORALS is to gain a deep understanding of existing approaches to recording visits (see Table 1). We will conduct site visits to the Ryan Family Practice, Ludington, MI; University of Texas Medical Branch Cancer Center Victory Lakes, League City, TX; and Barrow Neurosurgical Institute, Phoenix, AZ. Each of these sites has a unique approach to recording visits and thus important insight to share that will help guide the development of ORALS.

#### Purpose

**Figure 2:**
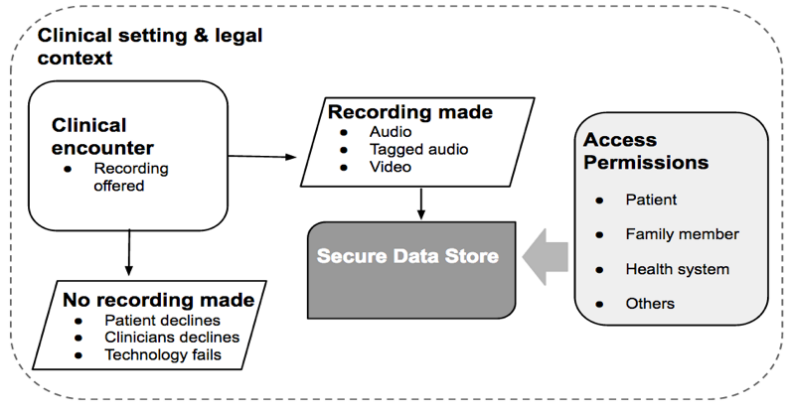
A conceptual path to recording, tagging, and sharing the clinical encounter.

Our purpose is to gain insight from pioneer clinics to guide the development of ORALS. In stages 2, 3, and 4, we will use case study findings to develop ORALS and test the software in a human-computer interaction laboratory and in clinic settings. The ic3d team and patient representatives will be involved in stage 1 by guiding the study design and interpreting findings. Figure 2 illustrates the critical steps, from the challenges of accurate physical recording to creating a secure data store for safe multiple use access.

**Table 1:** Case study research guide (informed by our previous research [53]).

Research questions	Data sources
RQ1. What are the technological aspects of successful recording software and user interface?	Interview (clinicians, patients, family members, software developers, clinic management and administrators); documentation (study reports, specifications of software and hardware), direct observation (use of recording technology)
RQ2. Why and how was the recording and sharing of clinical visits with patients adopted?	Interview (clinicians, clinic management and administrators, patients), documentation (policy documents, consent forms, survey data, publications), archival (proportion of clinicians who offer recording, log of recording use)
RQ3. How are recordings used?	Interview (clinicians, patients, family members, clinic management and administrators), documentation (survey data, publications), direct observation (use of recording technology)
RQ4. What is the added value of tagging recordings and what are the most important moments to tag?	Interview (clinicians, patients, family members, clinic management and administrators), documentation (survey data), archival (log of system use)

#### Design

We will adopt a multiple case design including 3 sites where recordings of medical visits are shared with patients [51]. Each recording system represents a case (ie, the unit of analysis). Embedded in each case will be clinicians who support recording and those who do not, patients and their families, and clinic management and administrative staff.

#### Settings

##### Clinic 1: Ryan Family Practice, Ludington, Michigan

This primary care practice consists of a clinician, medical assistant, secretary, and a 2000-patient panel, with 120 patient visits per week. Dr James Ryan and Kevin Perdue have developed an electronic medical record system called the small brain records project that supports contemporaneous tagging of audio recordings as Dr Ryan enters patient data (eg, medication change). These tagged encounters and audio recordings are shared with patients and caregivers via a secure Web portal. While Dr Ryan plans to record all of his patient visits, he currently records approximately 50% of patient visits.

##### Clinic 2: University of Texas Medical Branch, Cancer Center Victory Lakes, League City, Texas

The University of Texas Medical Branch (UTMB) Cancer Center in Victory Lakes consists of 14 clinicians who each treat approximately 40 to 50 patients per week. Dr Meredith Masel of the Oliver Center for Patient Safety and Quality Healthcare, UTMB, Galveston, TX, has pioneered the routine implementation of audiorecording in UTMB Cancer Clinics through the “Taking the Message and the Medicine Home” program. Patients are offered a digital device to record visits or are educated about recording and using their own device.

##### Clinic 3: Barrow Neurological Institute, Phoenix, Arizona

Barrow Neurological Institute (BNI) is a large neurological disease institute. Each of its 27 neurosurgeons treats 50 patients per week. Currently 10 clinicians use the Medical Memory video recording service, developed by Dr Randall Porter, to record the visit and share with patients via a secure Web portal.

#### Ethics

This research has received Institutional Review Board (IRB) approval from the Committee for the Protection of Human Subjects at Dartmouth College (Study #29380).

#### Participants

Participants will include patients, family members, clinical staff, management, and administrators. We will use key contacts to arrange interviews and include clinicians who have chosen not to offer patients recordings. Interviewees will be 18 years or older and able to communicate in English. We plan to conduct a minimum of 6 interviews per stakeholder group at each site until data saturation is reached [53].

#### Data Collection

##### Overview

Yin [51] identifies 6 primary sources of evidence for case study research. Using multiple sources of evidence per research question increases the precision of findings through a process of triangulation, taking different angles toward studying the same phenomena. Four of these sources can be used to answer the research questions described above (Table 1). A study database will be created using ATLAS.ti qualitative data analysis and research software (Scientific Software Development GmbH), increasing the reliability of findings by creating a chain of evidence from data collected, coding, and linkages to research questions [51]. Further details of theses sources are described below.

##### Semistructured Interviews

Semistructured interviews will be conducted and audiorecorded. We plan to conduct the majority of interviews over 5-day site visits. Our information technology (IT) team will conduct interviews with technology experts at each site. Topic guides have been developed with patient representatives and IT staff (Multimedia Appendix 1). All interviews will be transcribed by Acusis medical transcription service.

##### Documentation and Archival Sources

Relevant documents will be requested from clinic staff (eg, clinic recording policies, patient information sheets). We will liaise with the IT team to assess technical barriers (eg, interoperability challenges) and, where available, the proportion of patients with recordings, length of recordings, and playback frequency.

##### Direct Observation

We will observe the process from introduction to recording, its completion, and sharing with the patient.

#### Analysis

Audio recordings will be transcribed verbatim. Data analysis will take place simultaneously with data collection, which, in turn, will assist in the iterative development of the interview guides. We will use a framework approach to analyze the data. This approach consists of 6 steps: familiarizing ourselves with the data, identifying a framework, and indexing, charting, mapping, and interpreting the data [54,55]. The research questions will provide the analytic framework with thematic comparisons across cases. Identifying recurring themes across sites is a strength of the multiple case study design. Two researchers will apply an initial codebook, and they will meet and include new codes into a revised codebook before conducting a secondary assessment of interview data. Emergent codes will be added to existing codes. Codes, memos, and short narrative summations of data will be entered into ATLAS.ti.

### Stage 2: Open Recording Automated Logging System Design

#### Overview

Case study information will determine the scope and functionality of Open Recording Automated Logging System (ORALS) and the user interface designed using usability engineering [56].

#### Purpose

Usability engineering will provide iterative, formative feedback from ORALS target users. Vinter et al [57] reported that usability errors dominate 60% of software problem reports. Myers and Rosson [58] reported that about half of code development is devoted to user interface design. To mitigate these challenges, early stakeholder engagement in ORALS design before initial software development and iteratively thereafter is crucial.

#### Design

A member of the interaction design team will be embedded in stage 1 of the case study. We will analyze which moments during the clinic visit matter most to patients and their families (RQ4, Table 1) to arrive at a standardized set of tags for the user interface (eg, test result, treatment plan). This set will be categorized into terms such as specific medical conditions or treatments and topics such as treatment decisions and plans. Case study data will also support the development of a set of activity scenarios and associated tasks for participants to complete using prototypes. This will include tasks such as authenticating into ORALS to allow the secure linkage of recordings to patients, controlling recordings (eg, starting and stopping), finding a recent appointment, finding tagged information in the recordings, and other derived tasks.

Paper prototyping activities will guide early software development cycles by engaging potential ORALS users. This work creates an interactive interface by using paper, pens, markers, Post-It notes, and other media to mock up multiple screens and overlays for users [59]. As users complete tasks, a member of the design team responds by changing the paper interface screens and overlays. This prototyping process offers the benefits of getting users involved in the design early, when there is evidence that users are much more likely to offer feedback because the interface appears more malleable and capable of change.

The design team will develop a usability specification for the set of tasks and define the expected interaction paths and the amount of time to complete each task. The usability specification will be used as a baseline for determining changes. Survey questions will be used to collect demographics, preassessment of user expectations of the system, posttask design feedback, and postassessment of user impressions of the system.

We anticipate 3 rounds of ORALS usability evaluation with stakeholder users: (1) paper prototyping as described, (2) formative evaluation with an early software prototype midway in the software development cycle, and (3) summative evaluation at the end of the development cycle to understand how the ORALS will work in the field and guide final changes.

#### Settings

Facilitator-led paper prototyping activities will take place in person with prospective users in the human-computer interaction lab, ic3d lab, Geisel School of Medicine; observers will take notes. For formative and summative usability evaluation, activities can be conducted in person or facilitated remotely using Web conferencing tools. We will use a software usability evaluation product called Morae (TechSmith Corp), which supports screen recording of software use and a log of mouse and keyboard interactions. Each session will be designed to last about an hour.

#### Participants

We will work with a minimum of 6 potential users, patients, and clinicians in each design round. Participants will be identified both from local clinics and the in-clinic test site at UTMB. Participants will receive $25 for their participation.

#### Data Collection

Paper prototyping sessions will provide data through observational notes, think-aloud statements, and resulting design artifacts from changes in each session. Software evaluation sessions will collect performance measures, think-aloud statements, survey responses, and recordings of the user interactions with the system.

- Design artifacts: Modifications made to the design on the paper prototype will be photographed/scanned for review during analysis.
- Performance measures: Morae will capture time to task completion, successes, and failures.
- Survey responses: Morae will introduce and store survey questions during each task. Questions will focus on user roles and experience, ease of use for tasks, understanding of visual information, and known design tradeoff decisions.
- Screen and input recordings: Morae will produce a screen and audio recording of the session by task with an associated log of user input and any real-time codes entered by the facilitator.
- Observation: The facilitator and/or an observer will take notes during the session of any design-related events that take place during the user interactions with ORALS.
- Think aloud: Session participants will be instructed on how to think aloud during the session [60]. This will involve users verbally narrating their goals and plans for interacting with ORALS and reactions to the system’s responses to their input.

#### Analysis

The primary evaluation goal is to refine the user interface design, providing a layout, interface controls, and workflow for creating and reviewing recordings in ORALS. A member of the research team with expertise in user design (CG) will analyze the results of the paper prototyping sessions for themes. CG will also review recordings of the software session for usability issues, including deviations from the expected interaction paths. Finally, CG will analyze performance measures, coded sessions, and survey responses. Modifications and enhancements to the interface will be based on commonality of themes.

#### System Prototyping

After the first round of usability evaluation through paper prototyping, we will create a set of software requirements for the initial ORALS prototype, and we will modify and improve the user interfaces for the software prototype based on feedback from the second and third rounds of usability evaluation.

### Stage 3: Open Recording Automated Logging System Tagging Software

#### Overview

**Figure 3:**
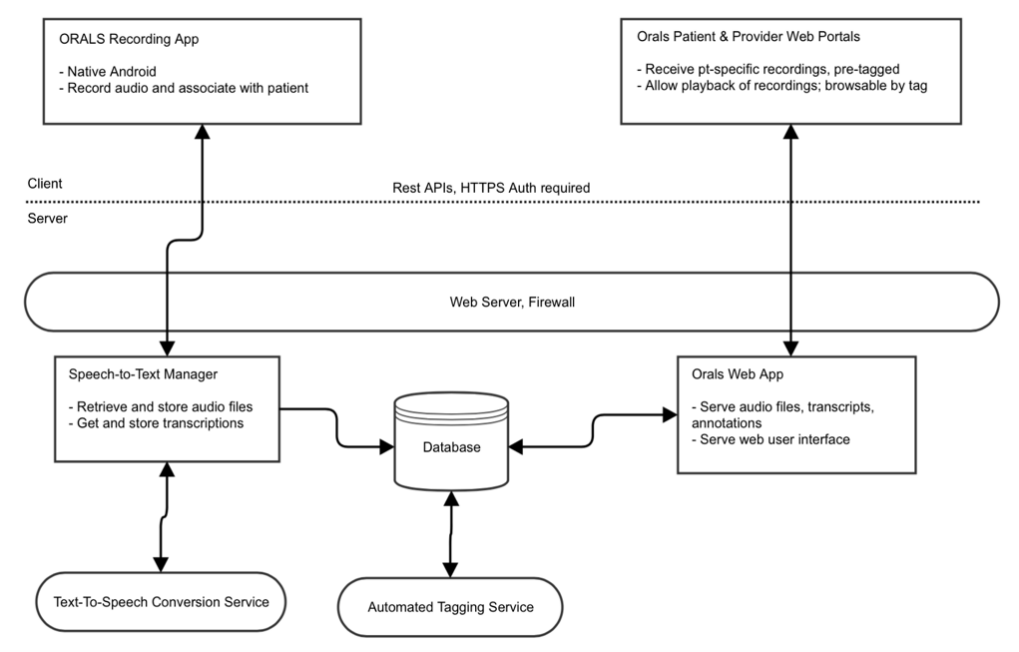
Open Recording Automated Logging System high-level architecture.

ORALS software will be developed and implemented as a secure Web-based system consisting of user interfaces (stage 2) supported by recording, transcribing, and automated tagging software (stage 3). User-facing portions (stage 2) of the software will be implemented in the Ruby on Rails Web development framework; the automated-tagging portion of the system will be written in the Python language (see Figure 3). The automated tagging software will have 2 components: speech recognition and tag identification. Speech recognition software has improved considerably in accuracy over the past 2 decades and is used in clinical settings for medical transcription [61]. We will use voice-to-text and text analytics approaches from IBM Research for speech-to-text transcription. For tag identification, we will use Weka [62] and SciKit [63], both open-source and widely used machine learning libraries.

#### Purpose

In this stage, our goal is to take the text output from the speech recognition component and develop machine learning methods that will support accurate tag identification.

#### Design

As noted in stage 2, we will formalize a list of standard tags that are either terms or topics. To capture the context of terms and phrases in text in our tagging system, first we apply a named-entity recognition system on input text to identify medical concepts and their corresponding classes. For this named-entity recognition task we will use Apache cTAKES information extraction framework [64] and Unified Medical Language System (UMLS) [65]. cTAKES is open-source software that identifies terms and phrases in text that correspond to UMLS concepts in high-level classes such as anatomy, symptom, procedure, disorder, and drug. To identify topics of interest within the transcriptions, we will use a supervised machine learning classifier based on a support vector machine (SVM) framework [66]. The text, extracted concepts, and their associated classes will be provided as input features to the SVM classifier. This classifier will identify the texts that are most related to a topic based on these features. If the accuracy of the SVM approach is less than 90%, we will try the alternative approach of Hidden Markov Model (HMM) [67]. We will implement the automated tagging software using the Weka, Natural Language Toolkit, and SciKit libraries in Python.

#### Transcription Evaluation

We have access to a set of 120 existing patient audio recordings from a primary care setting and will apply the speech-to-text software to translate the recordings into written text using voice-to-text and text analytics approaches from IBM Research. The accuracy of the automated speech-to-text transcriptions will be measured as the percentage of words correctly transcribed. This will be measured by first formatting all transcripts into a one-word-per-row format, then applying the OpenDiff tool to count the number of differences between the manual and software-generated transcripts. Percent correct will be expressed as the number of differences divided by the total number of words per transcript. We expect that an accuracy rate of 90% will be needed in order to have acceptable transcription performance for automated tagging.

#### Manual Tagging Evaluation

Four medical students will act as reviewers and will manually annotate the tags of interest in the text. Tags of interest will be derived through case study interviews with patients, caregivers, and clinicians. Students will work in pairs and will review 60 recordings each. An annotation guide will be created with a primary care clinician, who will also assist in training medical students in annotation. We will measure the Cohen's kappa coefficient between the 2 sets of reviewers and assess the interrater reliability, with a target kappa of >.8 [68]. Discrepancies will be resolved by consensus with other members of the research team.

#### Automated Tagging Evaluation

To automatically identify the tags related to terms, we will apply an SVM classifier to the transcriptions and their corresponding annotations using Apache cTAKES named-entity recognition method. We use 10-fold cross-validation to evaluate the classifier against the manual annotations (our reference standard). In this evaluation, we measure accuracy, precision, recall, and F1 score of our method. If the accuracy is below 90%, we will consider and evaluate the performance of other machine learning approaches for topic identification in text, such as an HMM.

#### Development

The usability testing and machine learning efforts in stages 2 and 3 will overlap so that the ORALS system can be developed and tested in an iterative agile approach. After paper prototyping is completed in stage 2, we will implement the initial Web-based prototype, which will include the manually tagged recordings, for the planned formative evaluation. For the planned summative evaluation, the ORALS system will include patient recordings that have been automatically tagged with terms and topics.

We will undertake 2 steps to ensure the privacy of the patient and physician participants who provided the initial recordings in our testing with other patient participants in usability evaluation. First, the project team will rerecord the transcription for the subset of recordings used in usability testing so that recording will be in the voices of project team members and not the original patient or clinician. Second, we will ensure that all protected health information defined by the Health Insurance Portability and Accountability Act (HIPAA) is removed from these recordings.

#### System Deployment

We will develop ORALS as a secure stand-alone Web-based application that can be accessed via the Internet from a home computer. The system will be hosted on HIPAA-compliant servers within Dartmouth College. For deployment and testing in stage 4, patient participants will access their individual recordings through authenticated log-in over an https secure online connection, ensuring the recording is linked to the correct patient. In addition, we will design audiorecording control interfaces that can be used from a native Android app on any compatible device. The interface will allow for the collection of the recording in the exam room via the Android device and the uploading of the recording in an encrypted manner directly to the ORALS database server.

### Stage 4: Open Recording Automated Logging System In Clinic

#### Overview

The final stage will involve rapid cycle testing of ORALS using the Model of Improvement approach, which consists of 3 improvement questions and Plan-Do-Study-Act (PDSA) cycles [69] (described below). The Centers for Medicaid and Medicare Innovations recommends this approach for testing innovative interventions. PDSA cycles are iterative small-scale tests of change consisting of a hypothesis for improvement (Plan), study protocol to implement and test the proposed improvement (Do), analysis and interpretation of the data (Study), and iteration of what to do next based on the study (Act) [69-72].

#### Goals

Our aim is to produce a version of ORALS that is widely accepted by clinicians and used by patients and their families. Each cycle will lead to refinements of ORALS, improving performance, usability, and acceptability. The introduction of ORALS will be considered a success if all of the following occur:

1. Patients are engaged in the system as evidenced by their use of ORALS. Use will be assessed by measuring (1) the proportion of eligible patients who consent to recording, (2) the proportion of consenting patients who access their recording (and playback frequency), (3) proportion of consenting patients who use tags, (4) proportion of patients who share their recording (and sharing frequency), and (5) time spent listening.
2. Accuracy of tagging is high. Patients will be asked to listen and indicate if ORALS accurately tagged the visit or not. We aim for 90% accuracy of topics.
3. Patient engagement increases. Patient engagement will be assessed using the Patient Activation Measure short form (PAM-SF, a 13-item patient-reported survey) (Multimedia Appendix 2) administered before and after recording playback [73].
4. Family members are better prepared to support the patient. We anticipate that family members will be better prepared to support the patient and will assess this using the Preparedness for Caregiving Scale (PCS, a 9-item caregiver-reported survey) (Multimedia Appendix 3) administered before and after recording playback [74,75].
5. ORALS is accepted by clinicians and patients. Acceptance will be assessed by semistructured interviews with end-users (patients, caregivers, and clinicians).

#### Setting

We will test ORALS in the 3 clinics of UTMB Family Medicine—Dickinson, Island East, and Island West—that are served by approximately 40 clinicians, with 15 to 25 daily patient visits per clinician. We will focus testing in one of these clinics, to be decided in conjunction with UTMB in year 2. UTMB has a dedicated quality improvement team with experience in rapid cycle testing that will assist in this stage.

#### Participants

Patients aged 18 years and older with access to the Internet who can communicate in English will be eligible for inclusion. Clinicians from the selected UTMB Family Practice clinic will be eligible for inclusion.

#### Plan-Do-Study-Act Cycles

##### Iteration Plan

Prior to PDSA cycles, recording hardware and ORALS software will be set up in the UTMB exam rooms and tested for audio quality. We will use written scripts from previously validated recordings read out loud in the exam room.

**Figure 4:**
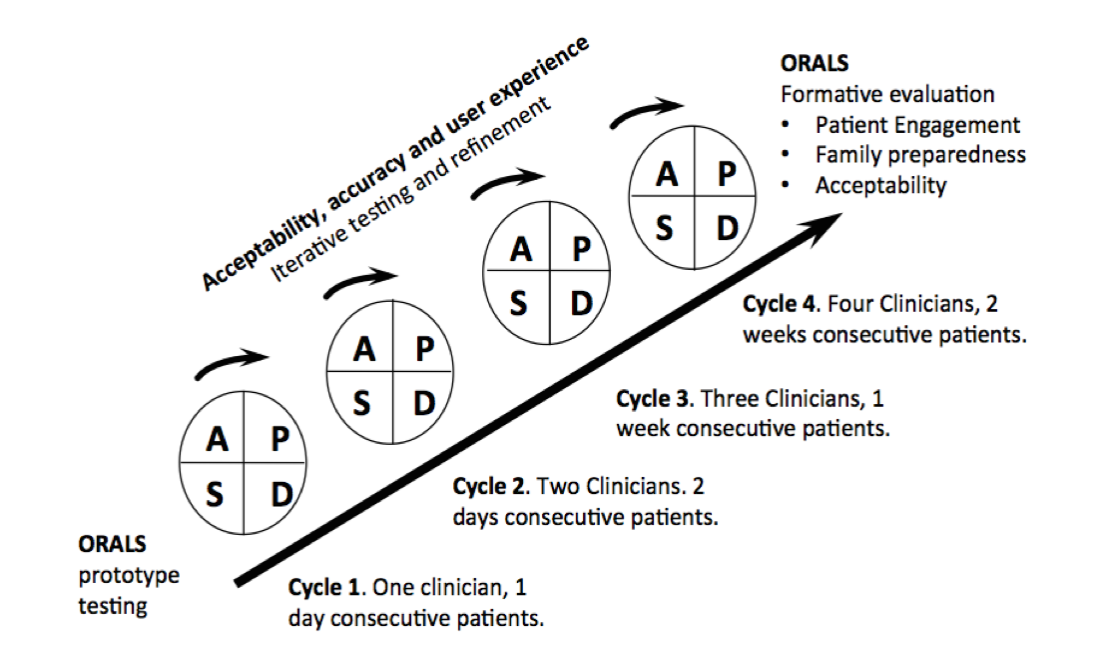
Plan-Do-Study-Act cycles: development of Open Recording Automated Logging System.

After testing, we will introduce ORALS for 1 day with consenting patients of a single UTMB clinician. A new clinician will be added per PDSA cycle, with 4 PDSA cycles in total (Figure 4). Each cycle will be used to refine ORALS, with results reported back to the IT team. PDSA cycles 1 and 2 will each take 1 month, and cycles 3 and 4 will each take approximately 2 months.

##### Assessment and Data Collection

Information on patient use of ORALS will be collected automatically within the system. This will include the number of patients who access their recordings, playback frequency, duration of use, time to first use, use of tags, and sharing frequency.

All users can leave feedback in an open text box. A selection of patients and family members (n=6-12) will be invited to take part in semistructured phone interviews within 1 week of their visit, each receiving $20 for participation. Clinician experience of ORALS will be assessed through semistructured interviews after each PDSA cycle.

Patient engagement will be measured with PAM-SF. Family engagement will be measured using the PCS. Surveys will be administered twice in the ORALS system, once prior to accessing recordings and again after ORALS use.

We will assess the accuracy of the tagging software by asking interviewed patients to relisten to their entire recording and verify each tag or identify missed tags, after which patients will be offered an additional $20.

#### Analysis

We will transcribe interviews and identify commonly reported issues and solutions in each cycle. A descriptive analysis of ORALS usage will be conducted with continuous data presented as means and standard deviations and categorical data as proportions and ranges. Paired *t* tests will compare PAM-SF and PCS scores before and after ORALS use. PAM-SF will be our primary outcome of interest. We will analyze PAM-SF on a continuous scale; scores range from 0 (low activation) to 100 (high activation). On average, interventions to promote engagement have required a 3- to 6-point change in PAM-SF to be a minimally important clinical difference [76,77]. With 200 patients, we will have 80% power to detect a 3-point change in PAM-SF with an alpha level of 0.05. We will aim for a minimum of 200 patients (25 patients per clinician per week) in the final cycle of PDSA testing. All data analysis will be conducted using Stata 14 (StataCorp LLC).

## Results

At the completion of this research, we will have developed an innovative platform for patients and their caregivers, consisting of easy-to-navigate recordings of clinic visits. We will implement a publication plan that includes a final project report and articles for peer-reviewed journals. In addition to this work, we will regularly report on our progress using popular relevant Tweet chats and online using our website, www.openrecordings.org. We will disseminate our work at relevant conferences (eg, Academy Health, Health Datapalooza, and the Institute for Healthcare Improvement Quality Forums). Finally, Iora Health, a US-wide network of primary care practices (www.iorahealth.com), has indicated a willingness to implement ORALS on a larger scale upon completion of this development project.

## Discussion

ORALS will offer patients and their families secure access to tagged recordings of health care encounters and will offer clinics a technically sound, interoperable, and secure system to facilitate routine recording. The proposed research is closely aligned with several of the strategies outlined in “A Roadmap for Patient and Family Engagement in Healthcare” [78].

### Patient and Family Preparation

The availability of recordings can “educate, prepare, and empower patients and families to engage effectively in their health and healthcare” [78]. ORALS can catalyze this increased engagement by providing recordings of clinic visits with key information, as identified by patients, tagged in an easy-to-navigate system and, in turn, creating an electronic library of their care history. For patients with low literacy, these audio recordings will be easier to navigate than written text. Additionally, caregivers often suffer from morbidity resulting from a perceived lack of self-efficacy related to the provision of care. ORALS will better prepare caregivers, often family, to engage in care by providing the information on the health condition and treatment plan and allow them to do this at distance. For example, a mother from Harrisburg, PA, can share a recording with her son in Los Gatos, CA.

### Transparency

“Nothing works well without transparency” [78]. There is no greater level of transparency and accountability than providing patients with access to recordings of clinical visits. This moves beyond giving patients access to the medical record, which still involves barriers for patients with low health literacy. Offering recordings of clinical visits appears to be the next step in transparency. Tagging recordings adds more value by providing structure based on the information that matters most to patients. Tagging is ubiquitous in today’s society bringing order and structure to masses of data—for example, the use of hashtags in Twitter groups tweets. ORALS will apply this same logic to health care recordings.

### Care and System Redesign

ORALS will facilitate information sharing and, in turn, the potential of greater care coordination across the health care system. ORALS will enable families to become a bigger part of the care team by allowing them secure access to health information. Recordings could also be shared with health professionals who receive a referral, increasing care integration. The fragmentation of health information technology is a significant barrier to sharing information within and between organizations, clinicians, and patients. ORALS will be an interoperable platform, offering a scalable solution designed to operate in any health information technology setting.

### Clinical and Leadership Preparation

Currently, many clinicians in training receive feedback based on a sample of visit recordings. Despite its value, this detailed feedback rarely occurs posttraining. ORALS could provide an opportunity for more routine performance assessment and feedback based on recorded visits in a safe and secure environment.

### Measurement and Research

The availability of detailed recordings would allow both patients and their families to provide feedback to clinicians on their performance. The availability of routinely collected recordings would also provide an opportunity for researchers, clinicians, and clinics to evaluate clinician performance.

The proposed ORALS aligns with the strategies outlined in “A Roadmap for Patient and Family Engagement in Healthcare” and has the potential to contribute to Gordon and Betty Moore Foundation’s Patient Care Program’s goals, and, thereby, the Triple Aim of better patient experience, better outcomes, and decreased cost. “We can't keep patients in the dark and then call them stupid for not having enough information” [78]. ORALS offers an opportunity to address this major information imbalance and bring patients and their families out of the dark.

### The Team

Our multidisciplinary team is uniquely positioned to successfully complete the proposed project. We have extensive experience in successfully developing technological solutions in health care and implementing novel interventions in primary care. We have a track record of engaging patient partners and other stakeholders as equal members of our research teams. Importantly, our patient partners and stakeholders have been engaged from the outset and represent a spectrum of perspectives including our target population, experience recording visits and accessing recordings as caregivers, clinician partners, knowledge of health IT regulatory and system requirements, and experience disseminating research findings to target audiences.

### Patient Engagement Activities

In addition to having patient partners as equal members of our research team, we will be holding “Lunch and Listen” exercises with patients from Dartmouth-Hitchcock’s volunteer support group. During these 90-minute sessions, 6 to 8 participants will have the opportunity to comment on the research design and share their views on recording. These exercises will be co-led by our 2 patient partners and occur on an annual basis.

### Conclusions

Upon the completion of this project we will have developed a novel recording system that will be ready for large-scale testing. Our long-term goal is for ORALS to seamlessly fit into a clinic’s and patient’s daily routine, increasing levels of patient engagement and transparency of care.

## Appendix

Multimedia Appendix 1 Interview topic guide.

Multimedia Appendix 2 Patient Activation Measure-Short Form.

Multimedia Appendix 3 The Preparedness for Caregiving Scale.

## Abbreviations

AVS: after visit summary
BNI: Barrow Neurological Institute
EHR: electronic health record
HIPAA: Health Insurance Portability and Accountability Act
HMM: Hidden Markov Model
ic3d: Informatics Collaboratory for Design, Development, and Dissemination
IRB: Institutional Review Board
IT: information technology
ORALS: Open Recording Automated Logging System
PAM-SF: Patient Activation Measure-Short Form
PSC: Preparedness for Caregiving Scale
PDSA: Plan-Do-Study-Act
SVM: support vector machine
UMLS: Unified Medical Language System
UTMB: University of Texas Medical Branch
