# Supplementary material for: Sharing Annotated Audio Recordings of Clinic Visits With Patients—Development of the Open Recording Automated Logging System (ORALS): Study Protocol
Source: JMIR Res Protoc. 2017 Jul 6;6(7):e121. doi: 10.2196/resprot.7735 (PMC5519830; doi:10.2196/resprot.7735)
Supplement: Multimedia Appendix 1 [file resprot_v6i7e121_app1.pdf]

Appendix 2. Interview Topic Guide

MANAGEMENT & STAFF INTERVIEW GUIDE

I. RECORDING IMPLEMENTATION

Q1.	What was the clinic's motivation to record and share clinic visits with patients?
Q2.	How long has your clinic been recording visits?
Q3.<br>●<br>●<br>●<br>●	What were/are the major challenges associated with introducing recordings?<br>Was there staff resistance?<br>Was there patient or caregiver resistance?<br>Were there legal concerns?<br>Were there technical challenges?
Q4.<br>●<br>●<br>●	What were/are the major facilitators easing the introduction of recordings?<br>Was staff accepting?<br>Was it patient and caregiver driven?<br>Did technology help?
Q5.<br>●<br>●	Do recordings fit into the clinic's workflow?<br>If so, how?<br>If not, why not?
Q6.	How does a patient walk through the recording process?
Q7.<br>●<br>●<br>●<br>●<br>●	How was the recording system developed?<br>Was it a package purchased from a technology company?<br>Was it set up by the clinic? If so, how?<br>Were patients, caregivers and/or clinicians involved?<br>Are the recording devices encrypted/secure?<br>Is there automatic playback on the device or does it need to be plugged into a computer?
Q8.<br>●<br>●	Is there anything you would change about the system?<br>The recording technology used in the clinic room?<br>The way recordings are shared with patients and caregivers?
Q9.<br>●<br>●<br>●<br>●<br>●	Where do you get the resources to support recordings?<br>IT<br>Hardware<br>Software<br>Expertise<br>Time
Q10.	How do you document which patients record their visits? Is it inputted into the EMR?
Q11.<br><br>●<br>●<br>●<br>●	Describe how you manage/protect the recorded data?<br>**PROBES:**<br>Is it on a secure server?<br>Are files encrypted?<br>Is access restricted among clinic staff?<br>How is PHI handled?

●	Is it HIPAA compliant?
Q12.	What are the concerns about the use of recordings? How have you overcome these?
●	Legal issues (or concerns) that have resulted from the use of recording? (If yes, explain more and how they were resolved)
●	Privacy concerns from patient or clinician?
Q13.	What are the benefits from the use of recordings in the practice?
●	Protection from litigation?
●	Patients better managing their own care?
●	Caregivers better prepared?

**II. RECORDING USE**	
Q14.	Do all of the clinicians in the clinic offer recordings to patients?
●	For those who don't, what are the reasons for clinicians choosing not to use of recordings?
●	For those who do (or if all do): what reasons do you believe have led to the use of recordings?
Q15.	How have patients responded to having access to their recordings?
●	Increased satisfaction?
●	Impact on frequency of clinic visits?
Q16.	How have caregivers responded?
Q17.	Do you use the recording data in any way (track recordings)? Are you considering using it in the future?
●	Review quality of clinician performance?
●	Litigation?

**III. TAGGING RECORDINGS**	
Q18.	If we used technology to highlight parts of the clinic visit, which parts would be most helpful for you?
●	Review of medical information?
●	Evaluation of test results?
●	Diagnosis?
●	Treatment options?
●	Treatment decision?
●	Visit summary?
Q19.	What might be the **benefits** to patients of recordings that are tagged? Explain.
●	What impact might tagging have on: patient understanding of health issues?
●	On patient ability to adhere to treatment plan?
●	On caregiver preparedness to assist patients?
Q20.	What might be the **drawbacks** to patients of recordings that are tagged? Explain.
●	Mis-tagged recordings (i.e. A part of the recording that is talking about the diagnosis is labeled as medication)

CLINICIAN INTERVIEW GUIDE

I. RECORDING IMPLEMENTATION

Q1.<br>a)<br>b)	Do your patients record their visits with you?<br>If yes, what proportion of patients record their visits? Of the patients who record, do they record all visits?<br>If no, what proportion do you offer recording to?
Q2.	Do you document which patients record visits?
Q3.	Are there groups of patients that recordings are particularly useful for? Why?
Q4.	Are there patients that recordings are particularly NOT useful for? Why?
Q5.<br>●	What was your motivation for starting to record visits?<br>Was it because of extrinsic factors, (i.e. leadership pressure, incentives) or intrinsic (i.e. wanted to improve your patient's care experience)?
Q6.	How long have you been allowing recordings of your visits?
Q7.<br>●<br>●<br>●<br>●	What were/are the major challenges associated with introducing recordings?<br>Was there staff resistance?<br>Was there patient or caregiver resistance?<br>Were there legal concerns?<br>Were there technical challenges?
Q8.<br>●<br>●<br>●	What were/are the major facilitators easing the introduction of recordings?<br>Was staff accepting?<br>Was it patient and caregiver driven?<br>Did technology help?
Q9.<br>a)<br>b)	Do recordings fit into the clinic's workflow?<br>If yes, how?<br>If no, why not?
Q10.	Does the use of recordings affect your practice style?<br>● The act of making the recording?<br>● The impact of patients or caregivers having a recording?
Q11. ●	Do you get resources to support recording? If so, what type?<br>IT, Hardware, Software, Expertise, Time, Etc.

**II. RECORDING USE**	
Q12.<br><br>●<br>●<br>●<br>●<br>●	Walk me through the recording process, from when the patient enters the exam room to sharing the recording with the patient.<br>Introducing the concept of recording (Every time? Even with patients who use the system?)<br>Starting the recording?<br>During the recording (practice as normal)?<br>Ending the recording<br>Sharing the recording
Q13.<br>●<br>●<br>●	Do you use the recordings patients make in any way? How?<br>Do you listen to them again? Yes or no = Why?<br>Share them with colleagues?<br>Would you use the recording if you have access?

Q14.	Can you imagine a way in which you would use recordings that you aren't doing yet?<br>● Sharing with colleagues for referral of a patient?<br>● Assessing quality of clinical performance?<br>● Recommending it as an adjunct to treatment?
Q15.	Do you use the recording data in any way (i.e. track recordings)? Are you considering using it in the future?<br>● Review quality of clinician performance?<br>● Litigation?
Q16.	Are there concerns about offering recording to patients?<br>● Legal issues (or concerns) that have resulted from the use of recording? (If yes, explain more and how they were resolved)<br>● Privacy concerns from patients?
Q17.	Are there benefits from the use of recordings in the practice?<br>● Protection from litigation?<br>● Patients are better managers of their own care?<br>● Caregivers are better prepared?
Q18.	How have your patients responded to having access to recordings?<br>● Are they more satisfied?<br>● Do they find it easy to use?<br>● Do they listen to recordings prior to subsequent visits?

**III. RECORDING PLATFORM**	
Q19.	Are there technical aspects of the recording system that facilitate its use by you?<br>● Process of recording the visit?<br>● Process of sharing the recording?<br>● Quality of recordings?
Q20.	Are there changes to the technology that may improve the recording system?<br>● Process of recording the visit?<br>● Process of sharing the recording?<br>● Clinician interface challenges?<br>● Patient interface challenges?<br>● Quality of recordings?

**IV. TAGGING RECORDINGS**	
Q21.	If we used technology to highlight parts of the clinic visit, which parts would be most helpful for you?<br>● Review of medical information?<br>● Evaluation of test results?<br>● Diagnosis?<br>● Treatment options?<br>● Treatment decision?<br>● Visit summary?
Q22.	What might be the **benefits** to patients of recordings that are tagged? Explain.<br>● What impact might tagging have on: patient understanding of health issues?<br>● On patient ability to adhere to treatment plan?<br>● On caregiver preparedness to assist patients?
Q23.	What might be the **drawbacks** to patients of recordings that are tagged? Explain.<br>● Mis-tagged recordings (i.e. A part of the recording that is talking about the diagnosis is labeled as medication)

Appendix 2. Interview Topic Guide

PATIENT (RECORDING USER) INTERVIEW GUIDE

I. RECORDING IMPLEMENTATION

Q1.	How did you find out that recordings are offered at your clinic?
●	In person
●	By email?
●	Your doctor?
●	Clinic staff?
●	Friend or family?
Q2.	What was your motivation for having your visits recorded?
●	Felt you were expected to?
●	Thought it would be useful for your care (how)?
Q3.	How long have you been recording visits? How many times have your visits been recorded?
Q4.	Walk me through the recording process, from when you arrive for your visit to when you receive the recording?
●	Does the clinician ask permission to record every time?
●	Are all of your visits recorded?
●	Are you aware when the recording begins, do you have to do anything (sign in to an account)?
●	Do you ever turn the recorder off during a visit? If so, when? And do you typically turn it back on?
●	When can you access the recording?
Q5.	Do you feel comfortable with the recording process?
a)	If yes, how long did it take you to get comfortable?
b)	If no, what makes it uncomfortable?
Q6.	Does recording affect how you speak with your clinician?
●	Are you more open, or closed?
●	How aware are you of the recording?
Q7.	Have you asked other healthcare providers to record visits?

II. RECORDING USE

Q8.	Have you listened back to a recording of your visit?
	**If yes,**
●	When do you listen to it?
●	Where and how do you listen to the recording(s)?
●	Why do you listen back?
●	How often do you listen to it?
●	How long do you spend listening back to a recording?
●	Can you walk me through that process of listening back to the recording?
Q9.	Have you listened back to a recording of your visit with a friend or family member?
●	Why
●	With whom?

Q10.	Have you shared your recording with anyone?
●	Why
●	With whom?
Q11.	Was it your idea to share the recording or was the idea of sharing a recording introduced to you?
Q12.	Do you have any concerns about recording your visit with the doctor?
●	Security issues?
●	Privacy issues?
●	Changes to your care?
Q13.	What have been the **benefits** of offering recordings in your care having recordings of your clinic visit?
●	Increased understanding of health?
●	Ability to share with others?
●	Capture bad experiences of care?
Q14.	What have been the **drawbacks** of having recordings of your clinic visit of offering recordings in your care?
●	Privacy concerns?
●	Too much information?

**III. RECORDING PLATFORM**	
Q15.	What was your experience like of using the recording system?
●	What was good?
●	What was bad?
●	Can you describe the sound quality?
●	Can you describe how you got access to the recording?
●	What was the process of sharing the recording like?
Q16.	What features of the recording platform do you like?
●	Look
●	Feel
●	Layout
●	Ease of use
●	Organization of recordings
Q17.	What features do you not like or would you change?
●	Look & Feel
●	Layout
●	Ease of use
●	Organization of recordings
Q18.	Are there any features missing from the platform?
Q19.	Are there technical aspects of the recording system that **facilitate** its use by you?
Q20.	Are there technical aspects of the recording system that are **barriers** to its use by you?
	Have you experienced any technical issues?
●	Sound quality?
Q21. ●	Access issues?
●	Sharing issues?
●	Difficulty to use?

**IV. TAGGING RECORDINGS**	
Q22.	If we used technology to highlight parts of the clinic visit, which parts would be most helpful for you?

●	Review of medical information?
●	Evaluation of test results?
●	Diagnosis?
●	Treatment options?
●	Treatment decision?
●	Visit summary?
Q23.	What might be the **benefits** to patients of recordings that are tagged? Explain.
●	What impact might tagging have on: patient understanding of health issues?
●	On patient ability to adhere to treatment plan?
●	On caregiver preparedness to assist patients?
Q24.	What might be the **drawbacks** to patients of recordings that are tagged? Explain.
●	Mis-tagged recordings (i.e. A part of the recording that is talking about the diagnosis is labeled as medication)

Appendix 2. Interview Topic Guide

PATIENT (NON-RECORDING USER) INTERVIEW GUIDE

I. RECORDING IMPLEMENTATION

Q1.	How did you find out about the process of recording?
Q2.	You've indicated that you don't currently record your visits, can you tell me why you have chosen not to use this service?
Q3.	Is there anything that would turn this into something you would like to use? Any features?
Q4.	

II. TAGGING RECORDINGS

Q5.	If we used technology to highlight parts of the clinic visit, which parts would be most helpful for you?
●	Review of medical information?
●	Evaluation of test results?
●	Diagnosis?
●	Treatment options?
●	Treatment decision?
●	Visit summary?
Q6.	What might be the **benefits** patients of recordings of recordings that are tagged? Explain.
●	What impact might tagging have on: patient understanding of health issues?
●	On patient ability to adhere to treatment plan?
●	On caregiver preparedness to assist patients?
Q7. ●	What might be the **drawbacks** to patients of recordings that are tagged? Explain.
●	Mis-tagged recordings (i.e. A part of the recording that is talking about the diagnosis is labeled as medication)

Appendix 2. Interview Topic Guide

FAMILY/CAREGIVER INTERVIEW GUIDE

I. RECORDING USE

Q1.	Has a family member shared a recording of their visit with you?
•	If yes, how did they do this?
•	Do you listen together in the same room, do you have your own access, or both?
Q2.	Have you listened back to a recording of your family member's visit?
•	<u>If yes,</u> what motivated you to listen back to this recording?
•	To understand patients health issues, treatment plan?
•	Reassure patient?
•	Felt obliged to?
Q3.	Do you listen back to every recording that is shared? (explain)
•	<u>If no,</u> can you talk a little more about why you have not listened back to your family member's recording?
•	Lack of time?
•	Lack of interest?
•	Can't make a difference?
Q4.	What have been the **benefits** of having access to recordings of these clinic visits?
•	Increased understanding of health?
•	Ability to share with others?
•	Capture bad experiences of care?
•	Help you provide better care?
Q5.	What have been the **drawbacks** of having access to recordings of these clinic visits?
•	Legal issues (or concerns) that have resulted from the use of recording? (If yes, explain more and how they were resolved)
•	Privacy concerns from patient or clinician?
Q6.	Do you have concerns about recordings being made of the doctor patient visits?
•	Security issues?
•	Privacy issues?
•	Changes to your loved one's care?

II. RECORDING PLATFORM

Q7.	What was your experience like of using the recording system?
•	What was good?
•	What was bad?
•	Can you describe the sound quality?
•	Can you describe how you got access to the recording?

III. TAGGING RECORDINGS

Appendix 2. Interview Topic Guide

Q8.	If we used technology to highlight parts of the clinic visit, which parts would be most helpful for you?<br>● Review of medical information?<br>● Evaluation of test results?<br>● Diagnosis?<br>● Treatment options?<br>● Treatment decision?<br>● Visit summary?
Q9.	What might be the **benefits** to patients of recordings that are tagged? Explain.<br>● What impact might tagging have on: patient understanding of health issues?<br>● On patient ability to adhere to treatment plan?<br>● On caregiver preparedness to assist patients?
Q10.	What might be the **drawbacks** to patients of recordings that are tagged? Explain.<br>● Mis-tagged recordings (i.e. A part of the recording that is talking about the diagnosis is labeled as medication)

SYSTEM DEVELOPER INTERVIEW GUIDE

I. PROCESS

Recording Environment	
Q1.	Where is the recording device (tablet) located?<br>• Is the device already in the consultation room<br>• Does the physician bring it<br>• Does patient bring it into the visit?
Q2.	How many people normally in the room, and are they participating?
Q3.	What is the actual sequence of events to start a recording?
Q4.	Is there a verification system in place? (i.e. this is the correct patient and it is uploaded to the correct medical record)
Q5.	How are recordings put on the server? When and by whom?

Equipment	
Q6.	What sort of equipment was used during recording?
Q7.	Were there any quality problems?
Q8.	What were common technical problems during recording?
Q9.	What is the actual sequence of events to start a recording?
Q10.	Have there been any battery life or disk-space problems?

II. RECORDING PLATFORM

Patient-facing	
Q11.	How do patients access recordings?
Q12.	What features does the platform contain?

Physician-facing	
Q13.	How do providers access recordings?
Q14.	What features does the platform contain?

III. SECURITY

Storage, transfer & access

Q15.	Once recording is made, how is it shared with patients?
Q16.	Are recording files encrypted?
Q17.	What happens to recordings after they are shared with patients (are they deleted or stored in the clinic system)?
Q18.	Where is the server? Prompt: can the device be locked up?
Q19.	How is data shared with patients & with family members?
Q20.	Are they able to download the data (recording)?
Q21.	What’s security like for the device (tablet) itself (while it contains recordings)?

IV. USAGE

Q22.	Any usage stats?
Q23.	How many users per account? (Just the patient, the patient’s family, etc.)
Q24.	What information do you collect on the person (meta data)? How is this collected?
Q25.	Who is generating the metadata?
Q26.	How soon after the visit are recordings accessible?
Q27.	For how long are recordings accessible?
Q28.	What’s security like for the device (tablet) itself (while it contains recordings)?
Q29.	How often do: 1) patients 2) clinicians **listen** to recordings?
Q30.	How often do 1) patients 2) clinicians **share** recordings?

V. SUPPORT

Q31.	Do you provide user support? If so, how much user support?
Q32.	What are common technical problems users have?
Q33.	Is documentation available for users (e.g. patient facing materials explaining or introducing the system; system specifications)?
Q34.	How do physicians get technical support and training?
Q35.	What are the common problems they encounter? Is there documentation of these problems?
