# Supplementary material for: Sharing Annotated Audio Recordings of Clinic Visits With Patients—Development of the Open Recording Automated Logging System (ORALS): Study Protocol
Source: JMIR Res Protoc. 2017 Jul 6;6(7):e121. doi: 10.2196/resprot.7735 (PMC5519830; doi:10.2196/resprot.7735)
Supplement: Multimedia Appendix 2 [file resprot_v6i7e121_app2.pdf]

Appendix 3.

PAM Questions

PAM_1	When all is said and done, I am the person who is responsible for taking care of my health	Disagree strongly	Disagree	Agree	Agree strongly	N/A
PAM_2	Taking an active role in my own health care is the most important thing that affects my health	Disagree strongly	Disagree	Agree	Agree strongly	N/A
PAM_3	I am confident I can help prevent or reduce problems associated with my health	Disagree strongly	Disagree	Agree	Agree strongly	N/A
PAM_4	I know what each of my prescribed medications do	Disagree strongly	Disagree	Agree	Agree strongly	N/A
PAM_5	I am confident that I can tell whether I need to go to the doctor or whether I can take care of a health problem myself	Disagree strongly	Disagree	Agree	Agree strongly	N/A
PAM_6	I am confident that I can tell a doctor concerns I have even when he or she does not ask	Disagree strongly	Disagree	Agree	Agree strongly	N/A
PAM_7	I am confident that I can follow through on medical treatments I may need to do at home	Disagree strongly	Disagree	Agree	Agree strongly	N/A
PAM_8	I understand my health problems and what causes them	Disagree strongly	Disagree	Agree	Agree strongly	N/A
PAM_9	I know what treatments are available for my health problems	Disagree strongly	Disagree	Agree	Agree strongly	N/A
PAM_10	I have been able to maintain (keep up with) lifestyle changes, like eating right or exercising	Disagree strongly	Disagree	Agree	Agree strongly	N/A
PAM_11	I know how to prevent problems with my health	Disagree strongly	Disagree	Agree	Agree strongly	N/A
PAM_12	I am confident I can figure out solutions when new problems arise with my health	Disagree strongly	Disagree	Agree	Agree strongly	N/A
PAM_13	I am confident that I can maintain lifestyle changes, like eating right and exercising, even during times of stress	Disagree strongly	Disagree	Agree	Agree strongly	N/A
