# Supplementary material for: Sharing Annotated Audio Recordings of Clinic Visits With Patients—Development of the Open Recording Automated Logging System (ORALS): Study Protocol
Source: JMIR Res Protoc. 2017 Jul 6;6(7):e121. doi: 10.2196/resprot.7735 (PMC5519830; doi:10.2196/resprot.7735)
Supplement: Multimedia Appendix 3 [file resprot_v6i7e121_app3.pdf]

Appendix 4.

The Preparedness for Caregiving Scale

YOUR PREPARATION FOR CAREGIVING

We know that people may feel well prepared for some aspects of giving care to another person, and not as well prepared for other aspects. We would like to know how well prepared you think you are to do each of the following, even if you are not doing that type of care now.

	Not at all prepared	Not too well prepared	Somewhat well prepared	Pretty well prepared	Very well prepared
1. How well prepared do you think you are to take care of your family member's physical needs?	0	1	2	3	4
2. How well prepared do you think you are to take care of his or her emotional needs?	0	1	2	3	4
3. How well prepared do you think you are to find out about and set up services for him or her?	0	1	2	3	4
4. How well prepared do you think you are for the stress of caregiving?	0	1	2	3	4
5. How well prepared do you think you are to make caregiving activities pleasant for both you and your family member?	0	1	2	3	4
6. How well prepared do you think you are to respond to and handle emergencies that involve him or her?	0	1	2	3	4
7. How well prepared do you think you are to get the help and information you need from the health care system?	0	1	2	3	4
8. Overall, how well prepared do you think you are to care for your family member?	0	1	2	3	4

9. Is there anything specific you would like to be better prepared for? ______________________________

____

____

____

____

MEAN SCORE of the number of items answered: __________

Reprinted with permission from author.

Stewart & Archbold (1986, 1994)
